# Implementation and first-year operating costs of an academic medical center-based syringe services program

**DOI:** 10.1186/s12954-021-00563-8

**Published:** 2021-11-19

**Authors:** Tyler S. Bartholomew, Hardik Patel, Kathryn McCollister, Daniel J. Feaster, Hansel E. Tookes

**Affiliations:** 1 Department of Public Health Sciences, Miller School of Medicine, University of Miami, 1120 NW 14th St, Miami, FL 33136 USA; 2 Department of Medicine, Miller School of Medicine, University of Miami, Miami, FL USA; 3 Department of Infectious Diseases, Miller School of Medicine, University of Miami, Miami, FL USA

**Keywords:** Cost, Syringe services programs, Implementation

## Abstract

**Background:**

Syringe services programs (SSPs) remain highly effective, cost-saving interventions for the prevention of blood-borne infections among people who inject drugs. However, there have been restrictions regarding financial resources allocated to these programs, particularly in the US South. This study aimed to provide cost data regarding the implementation and first-year operations of an academic-based SSP utilizing fixed and mobile strategies, including the integration of onsite wound care.

**Methods:**

We conducted a micro-costing study that retrospectively collected detailed resource utilization and unit cost data for both the fixed and mobile SSP strategies, including onsite wound care, from both healthcare and societal perspectives. A three-step approach was used to identify, measure, and value intervention costs, and cost components were categorized into implementation, variable program, and time-dependent costs. Sensitivity analysis was performed to examine the impact of SSP operational changes (i.e., needs-based distribution and opt-out HIV/HCV testing) on the cost-per-participant. Cost data we presented as overall cost and cost-per-participant adjusted to 2017 US dollars.

**Results:**

A total of 452 and 129 participants enrolled in fixed and mobile SSP services, respectively. The total cost associated with implementation and first year operations for the fixed site was $407,217.22 or $729.72 per participant and $311,625.52 or $2415.70 per participant for the mobile unit. The largest cost component for both modalities was time-dependent costs (personnel and overhead), while intervention materials (syringes, injection equipment, naloxone) were less than 15% of the total program cost.

**Discussion/conclusion:**

Implementation and operation of new SSP models continue to be low cost compared to treatment for the multitude of harms PWID face without access to evidence-based prevention. Future cost-effectiveness and cost–benefit analyses integrating a comprehensive SSP model within an academic institution, including onsite wound care and other medical services, will provide a more comprehensive understanding of this model, and state-level policy action must be taken to lift the prohibition of state and local funds for the implementation, sustainability, and maintenance of these programs in Florida.

## Introduction

Recent reports have indicated exponential increases in overdose deaths related to fentanyl [1] and stimulants [2], contributing to over 70,000 drug-related overdose deaths in the USA in 2019 [3, 4]. This trend has been further exacerbated by the COVID-19 with provisional overdose deaths climbing to over 90,000 in 2020 [5]. People who inject drugs (PWID) are at an increased risk of transmitting viral blood-borne infections, such as HIV and Hepatitis C (HCV) [6, 7], and acquiring bacterial and fungal infections, such as skin and soft tissue infections (SSTIs) and infective endocarditis [8] due to injection practices (e.g., receptive syringe sharing, re-using needles, improper cleaning of injection sites prior to injection). In 2018, PWID accounted for approximately 10% of all incident HIV infections in the USA [9] and have contributed to the increasing incidence of IDU-associated HIV outbreaks [10–15]. In addition, acute HCV infection has significantly increased since 2011, with 72% of the reported cases reporting IDU [16], making the prevention of these associated harms among PWID imperative.

One evidence-based, harm reduction intervention for preventing IDU-associated harms among PWID are syringe services programs (SSPs). The effectiveness of SSPs in reducing the transmission of HIV and HCV infections among PWID via the provision of sterile injection equipment has been overwhelming and well-documented [17–22]. SSPs have been highlighted as a cornerstone strategy under the “Prevent” pillar in the Ending the HIV Epidemic Initiative [23]. More recently, SSPs have performed as patient-centered medical homes and direct portals of entry into the health care system for PWID, allowing individuals to access HIV/HCV testing and treatment [24–27], linkage to substance use treatment [28, 29], low-threshold medications for opioid use disorder (MOUD) [30], overdose prevention through naloxone distribution and counselling [31], and medical services, such as primary care, vaccinations, and wound care [32, 33]. SSPs have also shown significant community-level impact, reducing the number of improperly discarded syringes in public spaces while not contributing to increases in crime and drug-related offenses [34, 35].

Implementation of SSPs has been shown to not only be an effective measure at averting HIV and HCV infections [7], but also a cost-effective public health strategy [20, 36]. These cost-effectiveness findings would be expected to translate to any setting serving an at risk population considering that a syringe costs a little as 7 cents [37], whereas the lifetime cost-savings from preventing one case of HIV is $389,359 [38], and the lifetime cost associated with HCV treatment is approximately $64,490 [39]. Recent data have also highlighted the vast cost-savings generated by SSPs with a one-year return on investment of $243 million in Philadelphia for HIV-related services only, with the majority of savings occurring in the public sector [20]. Additionally, the estimated cost of treating IDU-associated bacterial infections in the hospital was $380 million in one state (Florida) over one year, suggesting the actual cost-savings of SSPs are likely to be grossly underestimated for PWID-associated morbidity [40].

However, adoption and expansion of this evidence-based intervention has not been universal due to policy restrictions, opposition from law enforcement, and lack of political/financial support for program implementation and sustainability [41, 42]. In particular, states in the southern region of the USA, disproportionately affected by the HIV/AIDS epidemic [43, 44], have prohibited the implementation of these programs. In 2016, the Florida legislature passed the Infectious Disease Elimination Act (IDEA) authorizing the University of Miami to implement the first legal SSP in the state of Florida. Under this legislation, all state, county, and municipal money was prohibited from being appropriated to support this program. This critical piece of legislation was expanded in 2019 when all Florida counties were authorized to implement SSPs in their respective jurisdictions. With limited federal and state funding for implementation and sustainability of SSPs [45, 46] and previous research on SSP costs have focused on estimates and assumptions of service-payer perspectives only with a primary focus on fixed site operations [47], a more precise and accurate economic evaluation of newly implemented programs utilizing different modalities of intervention delivery with ancillary services, such as onsite wound care, is needed to inform policy makers and, more importantly, community stakeholders looking to implement SSPs in their respective jurisdictions. Given the change in SSP operations during the COVID-19 pandemic [48, 49] and future federal funding to support the implementation and expand the capacity of these programs, this study is particularly timely.

The primary aim of this study is to provide a detailed examination of healthcare-related and societal perspective costs using micro-costing methods associated with implementation and first-year operations of the IDEA SSP fixed and mobile programs in Miami, FL, including ancillary integrated services, such as wound care and overdose education and naloxone distribution (OEND).

## Methods

### Human subjects

This study was reviewed and approved by the University of Miami Institutional Review Board (IRB#20180483). Written consent was obtained from SSP staff that were interviewed and shadowed during primary data collection.

### Study setting

**Fig. 1:**
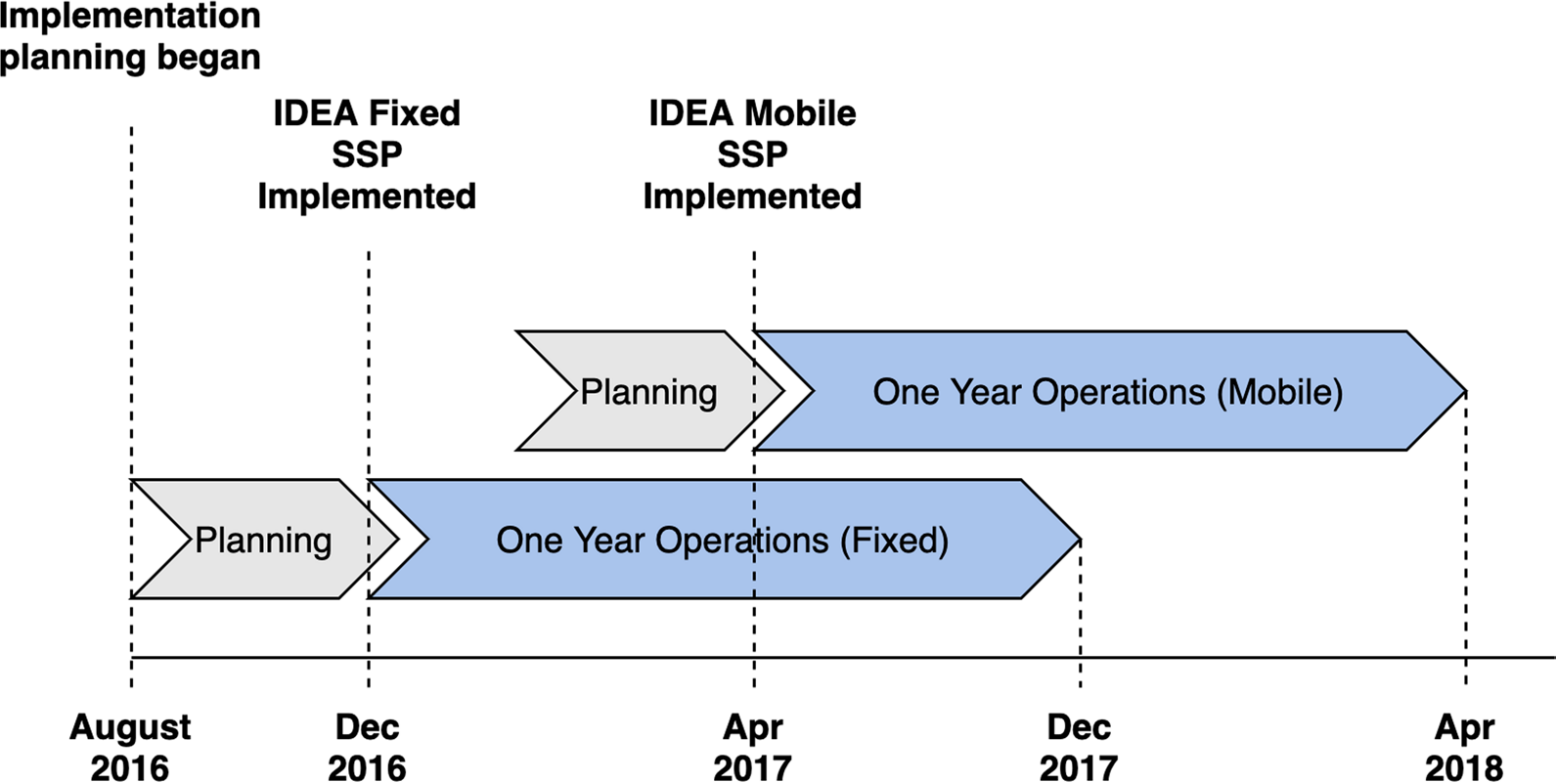
Timeline of fixed and mobile SSP implementation and operating costs timeframe

Programmatic data and grant expenditure reports were analyzed for the IDEA SSP fixed and mobile sites in Miami, FL during the first year of operation. The IDEA SSP is the first legal SSP in the state of Florida, administered by the University of Miami Miller School of Medicine, and primarily serves residents of Miami-Dade county. The IDEA SSP fixed location (i.e., shipping containers) opened on December 1st, 2016, and the IDEA SSP mobile unit, a renovated RV, was implemented April 17th, 2017, to target PWID with barriers to accessing services at the fixed site (Fig. 1). The fixed location operates Monday–Saturday for 33 h a week and the mobile unit operates Monday–Friday for 20 h a week.

### Program overview

The IDEA SSP fixed and mobile locations provide access to sterile injection equipment (e.g., syringes, needles, cookers, cottons, tourniquets, and sterile water) and other harm reduction supplies (e.g., naloxone, condoms) for PWID in addition to a venue for proper disposal of used syringes. At initial enrollment into the program, participants can receive HIV and HCV rapid testing alongside a 15-min demographic and behavioral assessment of current substance use, injection practices, and sexual behaviors. Participants are counseled on proper injection practices (e.g., cleaning site before injection, importance of not reusing syringes) and harm reduction strategies for reducing risk of drug-related overdose. Participants receive their HIV/HCV test results and are provided active linkage to HIV or HCV care if a participant is identified as reactive and not in care. Participants receive an initial installment of injection supplies and naloxone during the enrollment visit. After enrollment, participants are able to access the SSP services to exchange and receive injection supplies, additional naloxone doses, linkage to substance use treatment, and onsite wound care services through a free, student-run clinic. All programmatic data are collected, stored, and managed using REDCap, a secure, web-based platform for surveys and databases [50].

### Wound care clinic

In October 2017, the SSP staff partnered with the University of Miami Miller School of Medicine Mitchell Wolfson Sr. Department of Community Service to implement an onsite, medical-student run free clinic co-located at the IDEA SSP fixed location. The weekly walk-in clinic operates every Thursday from 4:00 to 8:00 pm and provides access to general health assessments, linkage to community resources, HIV/HCV testing, and management for skin and soft tissue infections, such as incision and drainage and wound care. All onsite physicians and medical students donate their time to the operation of the wound care clinic, and the wound care clinic is registered with the state of Florida as a Volunteer Health Care Provider Program (VHCPP). A more detailed description of the wound care clinic can be found elsewhere [33].

### Costing methodology

This study utilized a micro-costing approach [51] to identify and value the resources invested in implementation (i.e., startup phase) and first year operations of the IDEA SSP fixed and mobile sites, including the wound care clinic. Micro-costing involves the direct enumeration and costing of every input consumed in the treatment of a patient [52] and has been used as the foundation inputs for cost-effectiveness and cost–benefit analyses due to the precision of individual-level costing. Previous research has demonstrated that using micro-costing methods to measure important cost components can provide more reliable and valid estimates of total cost for healthcare-related services [53], and improves precision of cost estimates [51]. We applied this methodology to both the fixed and mobile sites from a healthcare and societal prospective. The micro-costing method was implemented in the 3-step process: (1) identifying resources used, (2) measuring resource use, and (3) valuing resources.

### Identifying resources used

In step one, we delineated all inputs that were used by each SSP modality to account for the cost of each component. The costs for delivering the intervention in each setting were partitioned into four categories: (1) implementation (i.e., start-up) costs, (2) variable program costs, (3) time-dependent program costs, and (4) societal costs. Implementation costs were defined as costs that were incurred before and during the initiation of the program. Implementation costs were further grouped into intervention material design (educational materials, participant ID cards, branding), administrative (hiring of staff), staff training, and property allocation (i.e., fixed site rental, purchasing a mobile unit). Implementation costs also included initial investment in program space and utilities, technical equipment (computers, iPads), office supplies and program furniture (e.g., chairs, tables, sharps containers, storage shelves). Variable program costs were defined as costs that were dependent on the number of participants served in the SSPs. This included harm reduction supplies and intervention materials (e.g., syringes, cotton, condoms), naloxone, personal sharps containers and HIV/HCV testing supplies. Time-dependent costs were defined as ongoing operations costs that were independent of the number of the participants that utilized program services. This included personnel time completing different service tasks (e.g., enrollment, daily exchange, outreach), administrative opportunity costs, phone rental, advertising, and facilities/utilities. Administrative costs are defined as costs associated with grant writing, advocacy, and program support in the form of in-kind program oversight (e.g., medical director, data analysis and management). For the broader societal perspective, we estimated the opportunity cost of volunteer effort (e.g., medical students) and time participants spent traveling to the program/accessing the program services.

### Measuring resources used

The next step in the micro-costing methodology is to measure the inputs of the program or intervention. To enhance accuracy of our cost data, we utilized numerous sources of data routinely collected by the SSP. Overall cost data were retrospectively aggregated and categorized from detailed expenditure reports and grant budgets filled out for each funding source supporting the IDEA SSP. Individual-level utilization, including number of visits, number of syringes exchanged, and distance travelled to the SSP were extracted from the IDEA SSP fixed and mobile enrollment and daily exchange databases and were used to calculate variable program costs. Mileage and fuel costs for the mobile exchange site are tracked in weekly logs by the mobile unit outreach coordinator. Hand-written logs are kept to track the hours spent by program volunteers. Some volunteers provide direct patient care via wound care (e.g., resident physicians), and their time was valued at the average hourly salary of support staff according to the Florida Volunteer Health Care Provider Program (VHCPP). Other program volunteer staff time (e.g., graduate/medical students performing administrative tasks) was valued at the average hourly salary of a student employee at the University of Miami.

To improve accuracy and to understand personnel time spent delivering components of the intervention (e.g., enrolling patients, performing HIV/HCV tests, conducting daily exchanges, data collection, supervision), individual, unstructured interviews were conducted with key program personnel, including the medical director, program director, fixed site operations manager, and the mobile site operations manager. In addition, SSP staff at both sites were shadowed by the study investigator for one week (each) to further corroborate the information gathered during the individual interviews.

### Valuing resources

In the final step, we quantified in dollars each type of resource consumed by multiplying a unit of a resource (e.g., an hour of staff time, one syringe) by its corresponding price weight or unit cost, and the results were summed to obtain total component-specific costs, total program costs by site, and cost per participant by site. Cost per participant per site was determined by dividing total program costs by the number of participants that enrolled during the first year of operation. A summary of the measurement units and the type of resource utilized is presented in Table 1. Program personnel time (program director, harm reduction specialist) were based on the actual yearly salary and corresponding fringe at the University of Miami. Intervention supplies (e.g., syringes, cookers, cottons) were estimated by multiplying the year-to-date number of syringes, cookers, cottons, and other injection supplies distributed by the purchased price per unit for said item. Harm reduction supplies were purchased through the North American Syringe Exchange Network (NASEN) Dave Purchase Project, and a detailed list of items and costs were provided by SSP staff to estimate per-unit costs. The cost of naloxone distribution was estimated by multiplying the actual number of naloxone 2-dose boxes provided by the wholesale price for one box. Monthly space rental costs were based on the actual rental rate for each of the shipping containers in which the fixed site SSP is housed, including monthly utilities (e.g., Wi-Fi, security, electricity). Fixed site property allocation (i.e., cost of purchasing land) was estimated by determining the land value at which the fixed site was placed, according to the Miami-Dade Property Appraiser. This cost element was not included in the final cost estimation; however, it was provided for local jurisdictions to understand the total cost of program implementation. In addition, the mobile unit was provided in-kind to the program; however, we estimated what the cost would be if the mobile were to be purchased under the same specifications as donated. The monthly costs of telephone rental and information technology (IT) support were based on the actual expenditures available in financial reports. All additional costs (furniture, supplies, advertising, brochures, staff uniforms, trailer/van modifications) were also based on the actual expenditures for these items.

**Table 1:** Resource utilization categories and cost measurement

Resource category	Measured units	Valuation
Personnel time (staff)	Hours	Hourly wage (including fringe)
Facility and utilities	Rental	Per month container cost
Training and intervention materials	Items	Purchased cost
HIV and HCV tests	Items	Purchased cost
Equipment (e.g., computers)	Items	Purchased cost
Furniture (tables, chairs, desks)	Items	Purchased cost
Office supplies (paper, folders)	Items	Purchased cost
Refreshments (coffee, water, food)	Items	Purchased cost
Phone rental	By month	Monthly bill
Patient transportation	Individual trips	Driving or public transit
Patient time	Hours	Hourly wage rate

### Societal costing

To understand costs from a broader perspective, one aspect of societal costs, i.e., the participant-level costs associated with having only one legal SSP for the entire state of Florida, we sought to estimate the individual-level time spent traveling and engaging in the SSP services. Due to the assumption that the majority of mobile intervention services are delivered where people are, we only estimated the opportunity costs associated with participants accessing services at the fixed intervention site. To estimate participant’s travel time and expenses, we used Google Maps to estimate their time and mileage (roundtrip) walking, taking public transit and/or driving between self-reported ZIP code of residence and the exact address of the fixed site location. As we did not know the addresses, we selected a central public space (e.g., park, post office, etc.) in a ZIP code and used that location as a starting point. In addition, for public transit costing, the number of buses and public transit trains were calculated and multiplied by the cost of roundtrip fare. For drive-related costing, we multiplied the miles traveled by the federal mileage rate ($0.58). Based on the binned categories for annual income ($0-$4999, $5000-$14,999, $15,000-$29,999, $30,000-$44,999, $45,000-$59,999, $60,000-$74,999, $75,000-$99,999, > $100,000), we took the midpoint of each income bracket to estimate the participants’ annual income. We then calculated the hourly wage rate for each participant by dividing these midpoint salaries by 2080, under the assumption that participants worked full-time. The estimated time for a participant (*i*) to travel via walking, driving, or public transit (*j*) was multiplied by the hourly wage rate times the number of SSP visits.

$$O_{{{\text{travel}}\,{\text{costs}}\left( {ij} \right)}} = ((HW_{{\left( {ij} \right)}} * {\text{SSP}}\,{\text{visits}}_{{\left( {ij} \right)}} *T_{{{\text{traveled}}\left( {ij} \right)}} ) + ({\text{SSP}}\,{\text{visits}}_{{\left( {ij} \right)}} * {\text{Cost}}_{{{\text{travel}}\left( {ij} \right)}} ))$$

For the intervention-associated opportunity costs representing volunteer staff time, we multiplied the number of SSP visits by the amount of time participants’ take to complete intervention tasks (enrollment, HIV/HCV testing, daily exchanges) that was elucidated during staff interviews and multiplied that outcome by the participant’s estimated hourly wage.

$$O_{{{\text{intervention}}\,{\text{costs}}\left( j \right)}} = ({\text{HW}}_{\left( j \right)} * {\text{SSP}}\,{\text{visits}}_{\left( j \right)} *T_{{{\text{intervention}}\,{\text{tasks}}\left( j \right)}})$$).

The total opportunity cost for each participant was estimated by adding the travel-associated costs and the intervention costs. For participants who reported experiencing homelessness, we estimated travel costs using walking-associated cost, while those who reported stable housing were assigned driving-associated costs.

### Sensitivity analyses

We performed one-way and two-way sensitivity analyses to examine the variation in total costs in key cost categories based on program policy changes. We assessed changes in total costs under the following scenarios: (1) changing from one-for-one syringe distribution to needs-based distribution (recommended best practice), (2) changing from opt-in HIV/HCV testing to routine opt-out HIV/HCV testing during enrollment and every 3 months thereafter and (3) implementing both policy conditions simultaneously. In order to estimate the theoretical number of syringes and injection equipment needed by all program participants over the first year, we multiplied the self-reported mean number of injections per day in the previous 30 days at enrollment by the total time in the program. Daily injection estimates were reported in binned categories (1–2, 3–4, 5–7, 8–10, 11–15, > 15), and estimates were generated based on the lower limit of each binned category multiplied by the number of days in the program for participants who had more than one exchange encounter post-enrollment. This estimate is based on the assumption that for every one injection, each participant should use one clean syringe, cooker, cotton, and sterile water.

$$T_{{{\text{injections}}\left( i \right)}} = \# {\text{injections}}\,{\text{per}}\,{\text{day}}_{\left( i \right)} *{\text{Time}}\,{\text{in}}\,{\text{program}}_{\left( i \right)}$$

The theoretical number of HIV/HCV tests performed was determined by multiplying the number of participants enrolled by the percentage of testing uptake if opt-out testing was implemented [25], based on the assumption that all participants received testing regardless of self-reported status. To estimate the number of HIV and HCV tests to be completed at routine quarterly assessments, participants’ time since enrolling in the program was divided into 3-month intervals to determine the number of theoretical tests that should have been administered during the first year of operation. Based on previously published seropositivity among fixed and mobile site clients [54, 55], we estimated the number of participants who would test negative during enrollment and would be recommended to receive testing during subsequent visits.

In addition to healthcare perspective sensitivity analyses, we performed one-way sensitivity analyses from the societal perspective to understand a lower-bound cost scenario representing participants’ time traveling and accessing services if they were to be paid the Florida state minimum wage ($8.10 for 2017).

## Results

During the first year of operation for the fixed and mobile SSP sites, a total of 581 participants (452 at the fixed location, 129 at the mobile unit) enrolled into the IDEA SSP. A breakdown of program-level characteristics, such as number of participants enrolled, number of syringes distributed, number of naloxone boxes distributed, and HIV/HCV testing can be found in Table 2, and the individual-level socio-demographics, substances used, injection-related behaviors and HIV/HCV status are presented in Table 3.

**Table 2:** Program-level data stratified by SSP modality

Characteristic	Fixed site (Dec 2016–Nov 2017)	Mobile site (Apr 2017–Mar 2018)
Number of participants enrolled	452	129
Number of quarterly assessments	90	7
Number of syringes disposed	89,494	19,842
Number of syringes distributed	76,958	19,160
Syringe return ratio	1.16	1.04
Number of Narcan distributed	554	214
Narcan per participant	1.23	1.66
Number of HIV tests conducted	241	43
HIV testing uptake	44.5%	31.6%
Number of HCV tests conducted	170	41
HCV testing uptake	31.4%	30.1%

**Table 3:** Descriptive characteristics of participants enrolling in fixed and mobile syringe service programs, Miami, FL

Characteristics	Fixed site (n = 452)	Mobile site (n = 129)
Age (median, IQR)	37 (30–46)	39 (31–47)
Biological sex (*n*,%)		
Male	347 (76.8)	82 (64.1)
Female	105 (23.2)	46 (35.9)
Race/ethnicity		
Non-Hispanic White	246 (56.6)	49 (40.8)
Non-Hispanic Black	15 (3.4)	23 (19.2)
Hispanic	174 (40.0)	48 (40.0)
Annual income		
< $14,999	199 (49.9)	65 (67.7)
> $15,000	200 (50.1)	31 (32.3)
Current housing status		
Experiencing unstable housing	159 (39.5)	59 (61.5)
Housed	244 (60.5)	37 (38.5)
Substances Injected in previous 30 days		
Heroin	377 (83.4)	77 (59.7)
Cocaine	94 (20.8)	34 (26.4)
Methamphetamine	49 (10.8)	5 (3.9)
Crack-cocaine	35 (7.7)	12 (9.3)
Speedball	91 (20.1)	17 (13.2)
Fentanyl	38 (8.4)	11 (8.5)
Average number of injections per day in previous 30 days		
≤ 2	112 (25.8)	18 (20.5)
3–4	135 (31.0)	24 (27.3)
5–7	93 (21.4)	23 (26.1)
8–10	54 (12.4)	13 (14.8)
> 10	41 (9.4)	10 (11.4)
HIV-positive (self-reported and tested)	40 (9.0)	12 (13.8)
HCV-positive (self-reported and tested)	202 (47.2)	52 (59.8)

### Total program costs and costs per participant

**Fig. 2:**
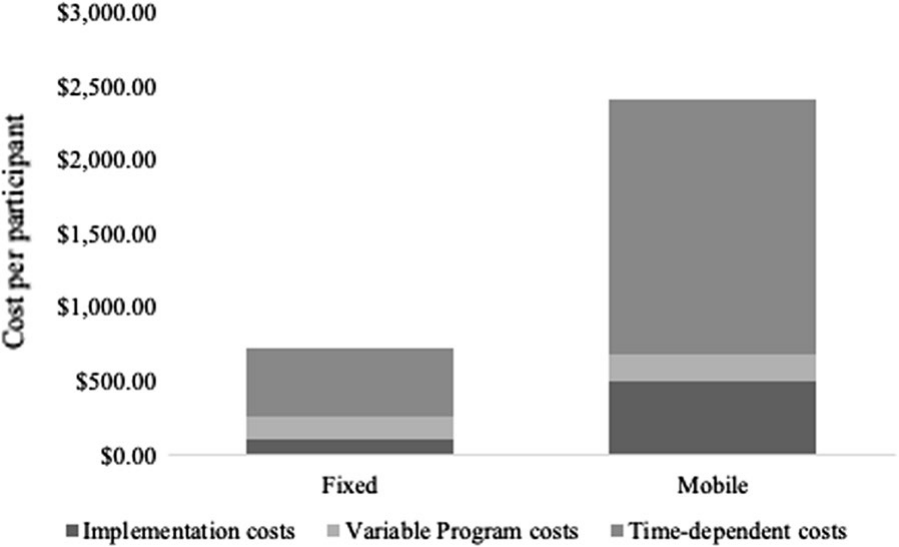
Comparison of cost per participant by cost component for the IDEA SSP fixed and mobile strategies

The total program cost and cost per participant stratified by SSP modality is presented in Table 3 in 2017–2018 US dollars. From the healthcare perspective (excluding the estimated societal costs), the cost to implement and operate the IDEA SSP fixed site during the first year was $407,217.22 ($729.72 per participant) and $311,625.52 for the IDEA SSP mobile site ($2415.70 per participant). From the societal perspective, the total opportunity costs associated with participants accessing services was $65,470.09 ($144.84 per participant) for the fixed site only. A breakdown of the total cost for each intervention site (fixed vs. mobile) is presented in Table 4. In addition, a graphical presentation of the percentage of the total cost per cost category by intervention site is presented in Fig. 2.

**Table 4:** Estimated total and per participant costs of implementing and first year operations of a fixed and mobile syringe service program

Cost category	Fixed site (N = 452)		Mobile site (N = 129)	
	Total costs	Cost per participant	Total costs	Cost per participant
**Implementation costs**				
Intervention material design	$2876.50	$6.36	$981.50	$7.61
Administrative	$16,133.32	$35.69	$12,141.68	$94.12
Staff training	$2821.78	$6.24	$1304.81	$10.11
Staff uniforms	$975.00	$2.16	$975.00	$7.56
Property allocation	$204,000.00*	$451.33*	$14,200	$110.08
Facility and utilities	$17,018.87	$37.65	$25,707.37	$199.28
Other utilities				
IT support for data collection	$4085.00	$9.04	$4085.00	$31.67
Phone purchase	$249.99	$0.55	$249.99	$1.94
Equipment				
Computers	$4116.00	$9.11	$2058.00	$15.95
iPads for data collection	$2388.00	$5.28	$2388.00	$18.51
Furniture	$1330.00	$2.94	–	–
Clerical supplies	$39.00	$0.09	$27.00	$0.21
Sharp containers (disposal)	$99.50	$0.22	$99.50	$0.77
**Total**	$52,132.96	$115.34	$66,709.89	$517.13
**Variable program costs**				
Intervention supplies				
Needles and syringes	$1231.33	$2.72	$306.56	$2.38
Cookers	$3847.90	$8.51	$958.00	$7.43
Cottons	$128.26	$0.28	$31.93	$0.25
Tourniquets	$5156.19	$11.41	$2567.44	$19.90
Crack pipe covers	$2360.00	$5.22	$590.00	$4.57
Condoms	–	–	–	–
Sterile water	$5894.98	$13.04	$1467.66	$11.38
Alcohol swaps	$969.67	$2.15	$241.42	$1.87
Paper bags	$246.29	$0.54	$54.82	$0.42
Personal sharps containers	$1756.84	$3.89	$716.43	$5.55
Narcan	$41,550.00	$91.92	$16,050.00	$124.04
Testing supplies				
HIV testing (point-of-care)	$2424.40	$5.36	$432.57	$3.35
HCV testing (point-of-care)	$3197.66	$7.07	$771.20	$5.98
Lancets	$65.76	$0.15	$13.44	$0.10
Total	$68,829.28	$152.26	$24,201.47	$187.22
**Time-dependent costs**				
Personnel time				
Participant enrollment	$18,765.00	$41.52	$7067.75	$54.79
Quarterly assessment	$6255.00	$13.84	$2355.75	$18.26
Daily exchanges	$35,580.00	$78.72	$23,557.50	$182.62
Outreach	$20,850.00	$46.13	$14,134.50	$109.57
Administrative	$77,375.36	$171,18	$48,650.04	$377.13
Administrative opportunity costs	$103,802.07	$229.65	$103,802.07	$804.67
Advertising	$17,475.14	$38.66	$17,475.14	$135.47
Rental space	$4036.80	$8.93	–	–
Phone rental	$599.88	$1.33	$599.88	$4.65
Wi-Fi hotspot	–	–	$232.80	$1.80
Refreshments	$321.73	$0.71	$321.73	$2.49
Sharps containers (disposal)	$1194.00	$2.64	$597.00	$4.63
Vehicle-related expenses				
Generator maintenance	–	–	$500.00	$3.88
Engine maintenance	–	–	$460.00	$3.57
Gas	–	–	$960.00	$7.44
Vehicle insurance	–	–	$2492.04	$19.32
Total	$286,254.98	$462.13	$223,206.20	$1730.29
Societal costs				
Intervention participants	$65,470.09	$144.84	–	–
Total program costs	$407,217.22	$729.72	$311,625.52	$2415.70
Total societal costs	$65,470.09	$144.84	–	–

^*^Cost not included in the calculation of total healthcare perspective costs

### Implementation costs

Implementation costs (infrastructure costs that are incurred regardless of patients receiving services) for the fixed and mobile SSP sites were $52,132.96 ($115.34 per participant) and $64,217.85 ($497.81 per participant), respectively. Of the total program costs per intervention site, implementation costs were the lowest cost component (17.2%) of the fixed site while they were the second highest cost component for the mobile site (30.9%). Within implementation costs, the largest percentage of costs was associated with facility implementation (e.g., shipping containers, security, fiber optics) at the fixed site (32.7%) and purchasing/renovating the vehicle for SSP functionality (62.1%) at the mobile site.

### Variable program costs

Variable program costs (costs dependent on the number of intervention participants) for the fixed and mobile SSP sites were $68,829.28 ($152.26 per participant) and $24,201.47 ($187.22 per participant), respectively. Of the total program costs per intervention site, variable costs were the second highest cost component for the fixed site (22.7%), while this was the lowest cost component for the mobile site (11.7%). For both the fixed and mobile sites, the largest costs within the variable program component were onsite overdose education and naloxone distribution (60.4% and 66.3%, respectively), while the provision of all harm reduction supplies (e.g., syringes, cookers, tourniquets, etc.) equated to 31.4% and 28.7% of the variable costs, respectively.

### Time-dependent program costs

Time-dependent program costs for the fixed and mobile sites were $286,254.98 ($462.13 per participant) and $223,206.20 ($1730.29 per participant), respectively. This cost component was the highest percentage of total program costs for both the fixed (70.3%) and mobile (71.6%) sites. The highest costs within the time-dependent cost component for both the fixed and mobile sites was administrative opportunity cost (36.3% and 46.5%, respectively).

### Wound care clinic costs

Between October 1^st^, 2017, and September 30^th^, 2018, 85 unique clients received services at the wound care clinic and a total of 114 visits were conducted across 45 clinic nights. The total cost of implementing and operating the wound care clinic for one year was $109,451.35 ($960.10 per visit), including the opportunity costs associated with the volunteer clinic staff. Excluding opportunity costs associated with volunteer physicians and medical students, implementation costs were $5238.36 ($45.95 per visit) and variable program costs of the medical care provided was $1892.92 ($16.61 per visit). A breakdown of the wound care costs is presented in Table 5, and a list of medical supplies ordered during the implementation phase can be found in “Appendix A”.

**Table 5:** Implementation and one-year operating costs of integrating an onsite wound care clinic

Cost category	Count	Per unit cost	Total cost	Cost per visit^e^
**Implementation costs**				
Phlebotomy chair	1	$800.00	$800.00	$7.02
Examine table	1	$1339.63	$1339.63	$11.75
Furniture	–	–	–	–
Administrative	–	–	$1271.50	$11.15
Medical supplies^a^	–	–	$1827.23	$16.03
Total			$5238.36	$45.95
**Variable program costs**				
HIV rapid test	46	$10.06	$462.76	$4.06
HCV rapid test	22	$18.81	$413.82	$3.63
Wound care^b^	31	$6.56	$203.36	$1.78
Incision and drainage^c^	17	$10.64	$180.88	$1.59
On-site Rx Antibiotics				
Bactrim (800 mg/160 mg)	280	$0.33	$92.40	$0.81
Doxycycline (100 mg)	42	$0.86	$36.12	$0.32
Snacks/refreshments	–	–	$503.55	$4.42
Total			$1892.89	$16.61
**Societal costs**				
Attending physician	1/clinic	$250.00/hour^d^	$45,000.00	$394.74
Resident physician	1/clinic	$26.00/hour^d^	$4680.00	$41.05
Medical students (onsite)	4/clinic	$25.43/hour^d^	$18,309.60	$160.61
Medical students (oversight)	6	$25.43/hour^d^	$34,330.50	$301.14
Total	–	–	$102,320.10	$897.54
Clinic total	–	–	$109,451.35	$960.10

^a^A detailed list of medical supplies that compromised this category can be found in “Appendix A”

^b^Wound care procedure cost breakdown: $2.16/cohesive bandage, $3.15/saline flush, $0.48/gauze, $0.02/alcohol pad, $0.15/antibiotic ointment

^c^Incision and drainage cost breakdown: $8.42/incision and drainage kit, $1.78/Chloraprep, $0.11/Lidocaine, $0.08/27G needle, $0.25/10 ml syringe

^d^Hourly wage estimated by the Florida’s Volunteer Health Care Provider Program (VHCPP)

^e^N = 114

### Sensitivity analyses

In order to understand the variation in the total cost estimates by intervention site, we conducted one-way and two-way sensitivity analyses for key cost elements. Particularly, we were interested in understanding how two evidence-based policies (needs-based syringe distribution and opt-out HIV/HCV testing) impacted the overall cost-per-participant estimates. Changing the syringe distribution policy from one-for-one to a needs-based model increased the cost per participant for both the fixed and mobile sites to $891.94 and $2658.49, respectively; however, this increase had a greater impact on the fixed site estimate (22.2%). If both sites had implemented routine, opt-out HIV/HCV testing at enrollment and subsequent testing every 3 months, the cost per participant for the fixed and mobile sites increased ($775.97 and $2481.30); however, this policy change had a significantly lower impact on the cost per participant (6.3% at the fixed site and 2.7% at the mobile site). If both policies were implemented simultaneously at both intervention sites, the cost per participant increased to $938 at the fixed and $2705 at the mobile sites (Table 6). Lastly, if participants who reported no annual income were assigned minimum wage, the societal cost would be minimally impacted ($149.93).

**Table 6:** Estimated per-participant cost by intervention modality in sensitivity analyses

Uncertainty examined	Fixed site	Mobile site
Base case	$729.72	$2415.70
One-way sensitivity analysis		
Changing syringe distribution policy to needs-based	$891.94	$2658.49
Routine opt-out testing from opening day	$775.97	$2481.30
Two-Way sensitivity analysis		
Changing syringe distribution policy to needs-based and implementing routine opt-out HIV/HCV testing when SSP was implemented	$938.05	$2704.68
Societal cost sensitivity analysis		
Base case	$144.84	–
One-way sensitivity analysis*		
Assigning minimum wage to participants who reported no income	$149.93	–

53 participant’s hourly wages were imputed with the minimum wage ($8.10)

## Discussion

While there is extensive literature highlighting the cost-effectiveness of SSPs [56–60], there is limited research examining the costs associated with program implementation by intervention delivery modality using micro-costing methods from routinely collected program data. This work builds on the data published in Teshale et al., 2019 by providing a more robust and precise estimation of the costs associated with implementation and first-year operations of a fixed and mobile SSP implemented in an urban setting established within an academic medical center. In addition, including the integration of onsite wound care services and overdose prevention through naloxone distribution, while expanding the costing perspective to include societal costs, is of notable importance. With the implementation and first-year operating costs associated with the fixed and mobile intervention venues totaling just over $700,000 and the lifetime cost of preventing one HIV infection over $380,000 [38], SSPs fundamentally provide communities impacted by the opioid and overdose epidemics an inexpensive, evidence-based strategy for HIV/HCV and overdose prevention. In more recent years, research has highlighted the utility of SSPs beyond their ability to “Prevent” new HIV infections. SSPs can offer HIV and HCV testing to “Diagnose” PWID with HIV [25], provide linkage to HIV and HCV care to “Treat” PWID living with HIV [61], and “Respond” to HIV outbreaks among PWID [62], suggesting SSPs have healthcare utility that surpass the historical scope limited to HIV and HCV primary prevention.

In this study, we found that the fixed site SSP venue had higher total implementation and first-year operating costs compared to the mobile site; however, the cost-per-participant for the fixed venue was substantially lower than the mobile modality. The lower cost per participant at the fixed site was likely due to limited hours and enrollment at the mobile site. However, previous research has demonstrated the need for mobile services to reach higher-risk PWID populations [55]. Given that the mobile unit engages higher rates of HIV and HCV infections, local policymakers and stakeholders may have higher willingness-to-pay (WTP) thresholds to prevent the spread of these infections. Programs should work to increase access to mobile SSP services to advance equity through appropriate hours of operation and targeted locations to optimize service delivery by conducting community-based needs assessments [63]. In addition, we found that the variable program cost-per-participant for the fixed and mobile sites were relatively similar ($152.26 and $187.22); however, the implementation and time-dependent (personnel and administrative time) costs were substantially higher per participant for the mobile site than for the fixed site. This finding suggests that the mobile unit, with limited operating hours and clients served, is more costly to implement and operate and this increased cost is not associated with the intervention supplies, including HIV and HCV testing.

Among variable program costs, harm reduction intervention supplies (e.g., syringes, cookers, cottons, sterile water, tourniquets and personal sharps containers) were only associated with 5.3% and 2.2% of total implementation and first-year operating costs for the fixed and mobile intervention sites, respectively. With an unprecedented amount of funding ($30 million) allocated for harm reduction programs in the American Rescue Act, including the use of these federal dollars for the procurement of syringes, jurisdictions looking to implement, operate, and sustain SSPs should work with policymakers to support intervention and non-intervention cost components (e.g., fixed-site land and rental space, purchase of a mobile unit, HIV and HCV testing, personnel time, and naloxone). In addition, due to the state-level prohibition of state and local funds in Florida, there was extensive resource allocation for administrative opportunity costs in order to support grant writing, fundraising, political advocacy, and program oversight. The extensive effort to change legislation and ensure sustainability of the program from a community and state level perspective should not be understated [64] and should be further examined in future SSP implementation costing.

The distribution of naloxone was the largest percentage of variable program-specific costs (> 60%) for both the fixed and mobile sites. Jurisdictions implementing the provision of naloxone should look to partner with community-based organizations or state/local health departments to provide this live-saving medication at no cost to SSPs. Research has shown that naloxone distribution through SSPs is cost-effective, and, when combined with linkage to substance use treatment and PrEP, is cost-saving [65].

In our sensitivity analyses examining the cost variation of needs-based syringe distribution and routine, opt-out HIV/HCV testing we found that these policy changes had minimal impact on the overall costs and cost-per-participant for both the fixed and mobile service modalities. The greatest increase in per-participant cost was seen with implementation of needs-based distribution at the fixed site, suggesting that the fixed site did not deliver sufficient intervention dosage (e.g., syringes and injection equipment) during the first year of operation [66]. However, this shortfall is due to ill-informed state-level policy that requires one-for-one distribution, which has been shown to be an ineffective method to reduce HIV risk among this population [67]. In addition, there was a minimal increase in per-participant cost when implementing a routine, opt-out HIV/HCV testing at enrollment and every 3 months thereafter. The utility of SSPs in HIV and HCV surveillance [62] is likely to be a cost-effective testing strategy in responding to outbreaks.

A major benefit for implementing SSPs at academic medical institutions is their ability integrate free medical services through a medical-student community service model, such as a wound care clinic [33]. The largest cost component for the onsite wound care clinic was personnel time (> 90%); however, these costs were not incurred by the SSP since all staff were volunteer medical students and attending physicians. With increasing rates of bacterial infections among this population leading to high economic cost [40], this type of wound care model may be highly cost-effective to prevent downstream infections and emergency room utilization.

### Limitations

There are several limitations that should be acknowledged when interpreting the findings of this study. First, this analysis only provides implementation and first-year operating costs for two different SSP service delivery modalities and does not examine the longitudinal changes in SSP operations and funding, including integration of linkage to care coordinators, telehealth services, and additional medical services, such as HAV and HBV vaccination, onsite PrEP, HCV treatment and behavioral interventions. As SSPs continue to integrate specialty services through low-barrier delivery to PWID, the longitudinal sustainability of these wrap around services needs to be considered, including the net economic impact in terms of societal costs vs. benefits. Second, since the overall program encompassed both the fixed and mobile unit venues, there is potential for misallocation of resources due to shared supplies, personnel, and administrative overhead between both locations. In addition, participants enrolled at either site were allowed to access syringes at either venue. However, there were minimal instances when this occurred, and the fixed and mobile sites collected individual-level data in separate databases, reducing the likelihood of misallocation of individual resources. Third, we were unable to estimate the cost of the land rental, as per this cost component was paid by the academic institution. Local jurisdictions should assess the cost associated with property rental and work with new programs to provide support for fixed site rental space for operations. Fourth, we relied on several assumptions when estimating the cost variation for needs-based distribution in the sensitivity analyses, assuming that substance use behaviors remained constant from when participants enrolled in the program. In addition, we did not consider participants’ engaging in substance use treatment or disengagement from using SSP services, potentially overestimating the “need” among program participants. Even so, we were able to use self-reported, individual-level data from the program to gather accurate estimates of the number of injections per day, number of SSP visits in the first year, and number of syringes distributed. Finally, we were unable to estimate the societal costs associated with the total time and effort to pass the initial SSP legislation in 2016 and the statewide expansion bill in 2019, which were critical to facilitate the implementation and operation of the program [64] and should be an area for future research.

## Conclusions

The findings from this study provide a robust and practical estimate for the implementation and first-year operating costs associated with a fixed and mobile SSP affiliated with an academic medical center. As additional programs are implemented across the state of Florida, implementers in local jurisdictions can utilize these findings to advocate for funding and establish expectations for resource requirements to implement and sustain these services going forward. In addition, since this analysis provides a framework for cost inputs that should be tracked by SSPs, new programs and future research should include prospective micro-costing methods to examine the longitudinal investment trends of SSPs, particularly as programs integrate wraparound services for their participants to ensure cost-effectiveness. Taken together, fixed and mobile site SSPs offer access to life-saving services for PWID at relatively low cost, and jurisdictions should consider providing funding opportunities for the implementation and sustainability of these evidence-based programs.

## Data Availability

Not applicable.

## Appendix A: Medical supply list and manufacturer for onsite wound care

**Table:**

Item	Cost	Unit	Cost/unit	Manufacturer	Item #
MooreBrand Sterile 4 × 4 gauze (12 ply)	$15.19	100	$0.15	Moore Medical	8252
MooreBrand Non-sterile 4 × 4 gauze (8 ply)	$11.99	200	$0.06	Moore Medical	12278
MooreBrand Non-sterile 2 × 2 gauze (8 ply)	$4.79	200	$0.02	Moore Medical	37336
Kerlix, Sterile Soft Pouch (4.5" × 4.1 yd)	$27.99	10	$2.80	Coviden	59713
Kendall Hypoallergenic Clear Tape	$22.89	12	$1.91	Coviden	87408
Bandaids Flexible Fabric (1" × 3")	$9.29	100	$0.09	Johnson and Johnson	13389
Coban Self-Adherent Wrap (tan 3" x 5yds)	$85.29	24	$3.55	3 M	35349
Telfa (3" × 4")	$29.39	100	$0.29	Coviden	80669
Xeroform (4" × 4")	$60.79	25	$2.43	Coviden	77549
Curity Plain Packing Strips (1/4" × 5 yds)	$70.68	12	$5.89	Coviden	65502
Saline Flush Syringe (10 ml)	$125	100	$1.25	B Braun	69606
Saline Irrigation (500 ml)	$80.82	18	$4.49	Baxter	15070
Betadine (4 oz)	$2.89	1	$2.89	Purdue Product LP	89390
Chloraprep One-step (1.5 ml)	$35.79	20	$1.79	B-D	91180
MooreBrand Triple Antibiotic Ointment (1.0 gm)	$26.49	144	$0.18	Moore Medical	82466
Lidocaine 1% (10 mg/ml, 20 ml)	$46.39	25	$1.86	Hospira Worldwide Inc	17392
Medi-Pak Performance Plus Surgical Skin Marker	$49.79	50	$1.00	–	13599
Wound Measuring Device	$2.09	50	$0.04	–	21593
MooreBrand Cotton Tipped Applicators (6" non-sterile)	$9.29	1000	$0.01	Moore Medical	69620
MooreBrand Suture Removal Kit	$124.50	50	$2.49	Moore Medical	82720
MooreBrand ER Lac Tray	$175.08	12	$14.59	Moore Medical	82767
MooreBrand I&D Tray	$187.80	20	$9.39	Moore Medical	82764
Lavender Nitrile Exam Glove (small)	$19.79	250	$0.08	Halyard Health, Inc	86591
Lavender Nitrile Exam Glove (medium)	$23.39	250	$0.09	Halyard Health, Inc	86592
Lavender Nitrile Exam Glove (large)	$19.79	250	$0.08	Halyard Health, Inc	86593
Fluidshield Fog Free Procedure Mask	$39.69	25	$1.59	Halyard Health, Inc	85851
SafetyPlus Polyethylene Gown	$89.69	75	$1.20	TIDI Products	16088
MooreBrand All Tissue Exam Gown	$33.49	50	$0.67	Moore Medical	77022
Emesis Basin	$2.99	10	$0.30		79440
MooreBrand Table Paper (White 21" × 225')	$46.19	12	$3.85	Moore Medical	76994
Syringe (Luer-Lok Tip 10 ml)	$50.78	200	$0.25	B-D	34325
Magellan Safety Needle and Syringe (20G)	$38.59	50	$0.77	Coviden	79647
Magellan Safety Needle (25G × 1")	$20.19	50	$0.40	Coviden	79633
Magellan Safety Needle (22G × 1")	$20.19	50	$0.40	Coviden	79629
Super Sani-Cloth Germicidal Disposable Wipe (6" × 6.75")	$11.09	160	$0.07	PDI	81831
MooreBrand Recycled Drape Sheets	$28.59	100	$0.29	Moore Medical	83413
Purell Advanced w/ Aloe Instant Hand Sanitizer (12 oz)	$77.09	12	$6.42	GOJO Industries	13503
MooreBrand True Metrix Pro Glucometer Test Strips	$24.79	50	$0.50	Moore Medical	26110
Curity Iodoform Packing Strips (1" × 5 yds)	$76.68	12	$6.39	Coviden	11234

## Abbreviations

SSP: Syringe Services Program
PWID: People who inject drugs
HCV: Hepatitis C
IDU: Injection drug use
SSTIs: Skin and soft tissue infections
IDEA: Infectious Disease Elimination Act
MOUD: Medications for opioid use disorder
